# Impact of integrated weed control methods on yield performance and sustainable weed suppression in sugar beet (*Beta vulgaris* L.)

**DOI:** 10.3389/fpls.2026.1743918

**Published:** 2026-02-11

**Authors:** Ömer Küçük, Olcay Bozdoğan

**Affiliations:** Department of Plant Protection, Agriculture Faculty, Malatya Turgut Ozal University, Malatya, Türkiye

**Keywords:** hoeing, integrated weed management, metamitron, sugar beet, sustainability, yield performance

## Abstract

Weed infestation remains a major constraint limiting the yield potential of sugar beet (*Beta vulgaris* L.) worldwide. Weeds compete with the crop for light, water, and nutrients, and if not effectively managed, they can cause severe yield and quality losses. Developing sustainable and efficient weed management strategies is therefore essential for maintaining crop productivity and reducing economic losses in sugar beet cultivation. This two-year field experiment (2020–2021) was conducted in Yeni Village, Doğanşehir District, Malatya Province, Türkiye, to assess the impact of various weed control methods on yield performance and weed suppression. The study employed a randomized complete block design with twelve treatments and four replications, including weedy and weed-free controls, herbicide applications, and combinations of herbicides with hand hoeing or power tillage. Yield components (total plant weight, root weight, leaf weight, root diameter, and plant length) and weed parameters (density, cover, fresh and dry weight) were recorded and statistically analysed. The highest yield was obtained from the weed-free control, followed by the integrated treatments combining pre-emergence herbicide (700 g L^-1^ metamitron) with one or two hand hoeings. These methods reduced the dry weight of the weeds by between 90.14% and 96.11% and increased yield by up to 365% compared with the weedy control. Herbicide-only treatments were less effective in sustaining yield and suppressing weeds. The findings emphasize that integrating chemical and mechanical control methods provides superior and more sustainable weed suppression, underscoring the importance of integrated weed management in sugar beet production systems.

## 1 Introduction

Sugar beet (*Beta vulgaris* L.) is one of the most important industrial crops globally, accounting for approximately 21% of total sugar production after sugarcane (USDA, 2023). Although several plant species contain sucrose, its efficient and economically viable extraction is limited primarily to sugarcane and sugar beet (Avcı, 2005). According to the Food and Agriculture Organization of the United Nations (FAO, 2023)), sugar beet is cultivated in 52 countries across nearly 4.6 million hectares, with Russia, the United States, France, Germany, and Türkiye collectively producing over half of the World’s total output. Average sugar beet yields reach about 60.1 t ha^-1^ globally, while France and Germany exceed 80 t ha^-1^. Türkiye ranks among the leading producers, with an average yield of 63.2 t ha^-1^, demonstrating strong potential for further improvement through agronomic innovation.

It is known that significant declines in production are occurring both globally and in our country, where the consumption of plant products is increasing due to diseases, pests, and weeds. The decline in crop yields due to weeds in plant production is at a level that cannot be ignored (Gökalp and Üremiş, 2015; Işık and Akça, 2018). In the production of major crops in the world (corn, wheat, sugar beet, potato, rice, cotton and soybean), yield loss due to diseases, pests and weeds is approximately 67%, of which 21% is caused by pests, 13% by diseases and 31% by weeds (Oerke et al., 1994). Weeds are known to cause an average of 5.8% of sugar beet losses. While this rate reaches 45% in Asian countries, it has been found to range from 6% to 40% in Turkey. Due to the slow germination of sugar beet seeds, weeds suppress sugar beet seedlings. In addition, it reduces the sugar content of sugar beets by 5-10%. In addition to production efficiency and sugar content, each of the processes of harvesting, transporting, and processing sugar beets in the factory to obtain sugar presents its own challenges (Güncan, 2000).

Despite its high yield potential, sugar beet production faces numerous biotic and abiotic challenges, among which weed competition remains one of the most critical. Weeds reduce yield and sugar content by competing for light, water, and nutrients, and they also act as alternative hosts for diseases and pests, exacerbating crop losses (Johnson et al., 1971; Gökalp and Üremiş, 2015). *Salsola kali* L. and *Atriplex* spp. harbor Curly Top Virus transmitted by leafhoppers, while *Alopecurus pratensis* L., *Sinapis arvensis* L., and *Portulaca oleracea* L. serve as hosts for nematodes such as *Heterodera schachtii* and *Meloidogyne* spp (Johnson et al., 1971). Yield losses due to weeds can vary widely depending on climatic and management conditions ranging from 6% to 40% in Türkiye and up to 45% in some Asian regions (Oerke et al., 1994; Işık and Akça, 2018). In addition to reducing root yield, weeds may lower sucrose content by 5–10% (Güncan, 2000).

Weed management is therefore crucial for maintaining the economic viability and sustainability of sugar beet cultivation. The crop’s slow germination and canopy closure make it particularly vulnerable to early-season weed interference. Effective control during this period is essential to prevent irreversible yield reductions (Işık and Akça, 2018). While herbicides have been the cornerstone of modern weed control, overreliance on chemical inputs raises concerns about environmental pollution, herbicide resistance, and production costs (Malaslı, 2010). Consequently, there is growing emphasis on Integrated Weed Management (IWM), which combines chemical, mechanical, and cultural practices to achieve effective, economical, and environmentally sound weed control (Kaya, 2012; Tursun et al., 2003).

Several studies have highlighted the benefits of combining herbicide applications with mechanical hoeing or reduced herbicide doses to sustain yield and minimize chemical dependence. Kaya (2012) reported that two tractor hoeings combined with thinning provided weed control efficiency up to 89.9%, comparable to full herbicide treatments, while reducing chemical use by nearly 70%. Such integrated systems not only enhance weed suppression but also contribute to sustainable agroecosystem management by improving soil health, reducing input costs, and maintaining long-term productivity. It has been reported that many annual and perennial weeds are found in sugar beet and their density is high (Tursun et al., 2003; Kordali and Zengin, 2008; Özkan and Kaya, 2008; Malaslı, 2010; Akça and Işık, 2016; Gökçe, 2018; Akar and Öğüt Yavuz, 2020; Büyükdemir and Kara, 2020; Üstüner, 2022; Yılar et al., 2022).

In this context, the present study aimed to evaluate the effectiveness of different weed control strategies including pre- and post-emergence herbicide applications, hand hoeing, and power tillage on weed suppression, yield performance, and sustainability in sugar beet. The findings are expected to contribute to the development of integrated weed management practices that enhance crop competitiveness, ensure high productivity, and promote sustainable sugar beet production under the semi-arid conditions of Eastern Türkiye.

## 2 Materials and methods

### 2.1 Study area and experimental conditions

This study was conducted as a two-year field experiment (2020–2021) in Yeni Village (38°12′N, 37°52′E), located in the Doğanşehir District of Malatya Province, Türkiye. The region lies within the Upper Euphrates Basin of Eastern Anatolia and is characterized by a continental climate, with hot, dry summers and cold, wet winters. The average annual temperature is approximately 12.6 °C, and long-term precipitation ranges between 288–497 mm (Meteoroloji Genel Müdürlüğü, 2022). During the experimental years, spring precipitation was slightly above the long-term average, which favored early seedling emergence and vegetative growth of sugar beet (**Table 1**).

**Table 1:** Meteorological data of Doğanşehir district*.

Month	2020					2021				
	Relative humidity (%)	Minimum temperature (°C)	Average temperature (°C)	Maximum temperature (°C)	Precipitation (mm)	Relative humidity (%)	Minimum temperature (°C)	Average temperature (°C)	Maximum temperature (°C)	Precipitation (mm)
1	74.90	-3.31	0.47	4.99	71.80	82.80	-4.77	-0.81	4.06	102.40
2	70.30	-2.91	0.86	5.14	67.60	66.70	-1.93	3.20	9.30	20.80
3	70.10	2.37	7.50	13.29	163.40	59.50	-0.36	5.41	11.48	61.60
4	57.30	4.42	10.97	17.42	15.40	50.50	6.09	12.97	19.55	7.60
5	51.10	9.08	16.43	23.12	61.40	39.10	9.20	18.80	27.09	2.20
6	45.50	11.38	20.67	28.65	13.20	40.20	11.88	21.29	29.46	1.40
7	38.20	15.94	25.70	34.56	0.00	35.30	16.78	25.95	34.19	0.20
8	36.60	12.91	23.13	32.70	0.60	40.90	14.89	24.42	33.71	5.20
9	43.00	11.97	21.88	32.23	7.20	45.10	10.05	18.99	27.66	6.80
10	44.20	6.07	15.39	25.35	0.40	50.30	4.79	12.48	20.76	4.60
11	70.80	0.65	6.05	12.31	71.00	65.00	2.21	8.53	16.37	1.80
12	82.00	-1.68	1.96	6.46	25.40	78.10	-3.11	0.47	5.30	73.60
Average	57.00	5.60	12.60	19.70	41.50	54.5	5.50	12.60	19.90	24.00
Annual precipitation: 497.40 mm (mm= kg m^-^²)						Annual precipitation: 288.20 mm (mm=kg m^-^²)				

*Data taken from MGM.

Soil samples were taken from the field where the experiment was conducted, from a depth of 0–20 cm, on April 1st of both years. Both soil samples were analyzed in the laboratory of Metalab. In 2020, the soil structure of the trial area was determined as clayey, pH value 7.72, lime content 30.34%, organic matter ratio 2.25%, phosphorus rate that the plant can absorb is 23.41 ppm, and potassium amount that the plant can absorb is 91.04 ppm, while in 2021, the soil structure was determined as clayey, pH value 7.81, lime content 19.22%, organic matter ratio 2.64%, phosphorus rate that the plant can absorb is 27.44 ppm, and potassium amount that the plant can absorb is 95.18 ppm.

### 2.2 Experimental design and treatments

In both years, the land was cultivated with a rotary plough and on April 10, a base application of a compound fertilizer called Beet NPK (12-30-12) was made at a rate of 150 kg per hectare in both years, and a fertilizer called urea 46% N (46) was applied at a rate of 180 kg/ha before planting. After the fertilizer spreading process, a cultivator was withdrawn once on April 11 in both years to mix the fertilizer and prepare the seed bed. After waiting two days, combine harrowing tillage was applied. Later, on April 15 in both years, sugar beet sowing was done with a 5-row sowing drill. Sowing was done in both years, with a 45 cm spacing between rows and a 15 cm spacing between rows. The plants were then thinned to 90,000 plants per hectare. In both years, the Aranka variety, treated with the active ingredients thiamehoxam + thiram + hymexazol from KWS, was planted.

Plots were created on April 17, taking into account the sowing order. Applications began in the trial plots on April 20, according to the study topics given in **Table 2**. The experiment was arranged in a randomized complete block design (RCBD) with 12 treatments and four replications. A design was created using 12 different weedy control methods, including weed control and weed-free control, to be applied in the experiment. These different research topics were applied in five different periods, as shown in **Table 3**. Counts were taken at 15-day intervals following sugar beet emergence. In addition to the percentage coverage of weeds, the effect of weeds on sugar beets was calculated at the end of the trial according to the criteria given below. In this study, one or more of the methods used in sugar beet cultivation and weed control from past to present were used in order to determine which method or methods were most effective in sugar beet. Trials were conducted using hand hoeing, pre-emergence herbicides, post-emergence broadleaf herbicides, post-emergence narrowleaf herbicides, power tiller, and a combination of these methods. Herbicides were applied at recommended doses. The combination of the above-mentioned herbicides and other methods, along with the abbreviations used in creating the experimental design, are provided (**Table 2**).

**Table 2:** Applications in the trial design. their contents and abbreviations.

Application codes	Applications
T1	Weedy control
T2	Weed-free control
T3	Pre-emergence herbicide application (700 g L^-1^ metamitron)
T4	Post-emergence broad and narrow leaf herbicide mixture application (100 g L^-1^ clopyralid, 100 g L^-1^ propaquizafop)
T5	Pre-emergence herbicide application (700 g L^-1^ metamitron) + Post-emergence broadleaf herbicide application (100 g L^-1^ clopyralid)
T6	Pre-emergence herbicide application (700 g L^-1^ metamitron) + Post-emergence narrow-leaf herbicide application (100 g L^-1^ propaquizafop)
T7	Pre-emergence herbicide application (700 g L^-1^ metamitron) + Hand hoe once
T8	Pre-emergence herbicide application (700 g L^-1^ metamitron) + Hand hoeing twice
T9	One-time hand hoe + Post-emergence broad and narrow leaf mixture herbicide application (100 g L^-1^ mlopyralid, 100 g L^-1^ propaquizafop)
T10	Hand hoeing twice + Post-emergence broad and narrow leaf herbicide mixture application (100 g L^-1^ clopyralid, 100 g L^-1^ propaquizafop)
T11	Pre-emergence herbicide application (700 g L^-1^ metamitron) + Power tiller once
T12	Pre-emergence herbicide application (700 g L^-1^ metamitron) + Power tiller twice

**Table 3:** Sugar beet development stages and applications in the experimental design.

Application codes	Sugar beet development stages				
	Pre-Exit period	Cotyledon stage (BBCH:10)*	2-4 Leaf stage (BBCH:12-14)	4–6 Leaf stage (BBCH:14-15)	8–10 Leaf stage (BBCH:19)
T1				Thinning	
T2		Hand hoe	Hand hoe	Thinning + Hand hoe	Hand hoe
T3	700 g L^-1^ metamitron			Thinning	
T4			100 g L^-1^ clopyralid 100 g L^-1^ propaquizafop	Thinning	
T5	700 g L^-1^ metamitron		100 g L^-1^ clopyralid	Thinning	
T6	700 g L^-1^ metamitron		100 g L^-1^ propaquizafop	Thinning	
T7	700 g L^-1^ metamitron		Hand hoe	Thinning	
T8	700 g L^-1^ metamitron		Hand hoe	Thinning	Hand hoe
T9		Hand hoe	100 g L^-1^ clopyralid 100 g L^-1^ Propaquizafop	Thinning	
T10		Hand hoe	100 g L^-1^ clopyralid 100 g L^-1^ propaquizafop	Thinning	Hand hoe
T11	700 g L^-1^ metamitron		Power tiller	Thinning	
T12	700 g L^-1^ metamitron		Power tiller	Thinning	Power tiller

(BBCH:10, Sugar beet cotyledon leaf stage; BBCH:12-14, Sugar beet 2–4 leaf stage; BBCH:14-15, Sugar beet 4–6 leaf stage; BBCH:19, Sugar beet 8–10 leaf stage).

The most commonly used and preferred methods in many regions were used in the experimental design. The herbicides used in the trial were selected based on their widespread use in root vegetables, their diverse mechanisms of action, and their importance within integrated weed management programs. The integrated use of herbicides is important in terms of increasing weed control effectiveness, reducing reliance on a single control method, and contributing to sustainable weed management. The application periods were chosen to reflect the most appropriate times for weed control (**Table 3**).

Each plot measured 5 × 4 m (20 m²), with 45 cm row spacing and 15 cm intra-row spacing, corresponding to approximately 100 plants per plot. The total area of the trial plots was 960 m². The total trial area was 1416 m². The trial plots were established 10 meters inward to prevent drying from the edges. A 1 m gap was left between each plot and each replicate to prevent the treatment from being applied to the upper, lower, and adjacent plots. In the trial, spraying was carried out using a fan-shaped T-jet nozzle and a pulverizer with a water flow rate of 300 liters ha^-1^ at 3 atm pressure. The first pre-emergence spraying was carried out on April 20. After the first application, the pre-emergence spraying was performed, the sugar beet was allowed to emerge from the soil surface. Sugar beet emergence began on April 25th. From that date onward, four random 1-square-meter frames were placed within each 20-square-meter plot, and these frames were fixed. A total of 192 fixed 1-square-meter frames were created within 48 plots. Sugar beet emergence was completed on April 30th. After the planting of sugar beets in 2021, it was observed that there were delays and incomplete emergencies in sugar beets due to lack of rain (**Table 4**).

**Table 4:** Applications and dates for sugar beet in 2020-2021.

2020 application dates	Applications made	2021 application dates	Applications made
April 15. 2020	Sugar beet planting was done	April 15. 2021	Sugar beet planting was done
April 20. 2020	Pre-emergence applications	April 20. 2021	Pre-emergence applications
April 30. 2020	Sugar beet and weed emergence	April 25. 2021	Sprinkler irrigation was done
May 1. 2020	1nd count was done	May 5. 2021	Sugar beet and weed emergence
May 2. 2020	Cotyledon application was performed	May 6. 2021	1nd count was done
May 5. 2020	Top fertilizer application	May 6. 2021	Top fertilizer application
May 16. 2020	2nd count was done	May 7. 2021	Cotyledon application was performed
May 17. 2020	2–4 leaf stage applications	May 10. 2021	1nd Irrigation was done
May 23. 2020	Copper and humic acid application	May 20. 2021	Copper and humic acid application
May 25. 2020	Thinning in 4–6 leaves	May 21. 2021	2nd count was done
May 26. 2020	1nd Irrigation was done	May 22. 2021	2–4 leaf stage applications
May 31. 2020	3nd count was done	May 30. 2021	Thinning in 4–6 leaves
June 7. 2020	8–10 leaf stage applications	June 1. 2021	2nd Irrigation was done
June 15. 2020	4nd count was done	June 6. 2021	3nd count was done
June 20. 2020	2nd Irrigation was done	June 7. 2021	8–10 leaf stage applications
June 30. 2020	5nd count was done	June 21. 2021	4nd count was done
July 10. 2020	3nd Irrigation was done	June 22. 2021	3nd Irrigation was done
July 15. 2020	6nd count was done	July 6. 2021	5nd count was done
July 30. 2020	7nd count was done	July 10. 2021	4nd Irrigation was done
August 1. 2020	4nd Irrigation was done	July 21. 2021	6nd count was done
August 15. 2020	8nd count was done	August 5. 2021	7nd count was done
August 30. 2020	5nd Irrigation was done	August 6. 2021	5nd Irrigation was done
September 15. 2020	Weed weighing was done	August 20. 2021	8nd count was done
October 1. 2020	Sugar beet harvest	August 26. 2021	6nd Irrigation was done
		September 5. 2021	Weed weighing was done
		September 20. 2021	Sugar beet harvest

Flood irrigation was used as the irrigation method for sugar beet in 2020. In 2021, due to lack of rain and sparse emergence, sprinkler irrigation was used once, followed by flood irrigation for subsequent irrigation. The practices used in the sugar beet trial were counts, irrigation, weed weighing and measuring, sugar beet weighing and measuring, and harvesting (**Table 4**).

### 2.3 The parameters investigated in the experiment are

#### 2.3.1 Sugar beet total weight

Twenty random sugar beets were harvested from each plot, free from edge effects, and weighed, ensuring that both leaves and roots were intact. The total weight of a plant was then calculated by averaging the 20 plants. The results were multiplied by the number of plants per hectare to calculate the total weight per hectare.

#### 2.3.2 Root yield in sugar beet

After removing the leaves from the sugar beets, only the root portion was weighed. The root weight of a plant was then calculated by averaging 20 plants. The results were multiplied by the number of plants per hectare to calculate the root weight per hectare.

#### 2.3.3 Sugar beet green parts weight

To determine the weight of the green parts, the weight of the cut leaves was taken. The average of 20 plants was then calculated to determine the green part’s weight of a plant. The results were multiplied by the number of plants per hectare to calculate the green parts’ weight per hectare.

#### 2.3.4 Sugar beet root diameter

Diameter was measured at the thickest point between the neck and stem of the sugar beet on the lump. The root diameter of a plant was then calculated by averaging 20 plants.

#### 2.3.5 Sugar beet length

After measuring the total length of the sugar beet, the leaf and root lengths were measured separately. The average length of the 20 plants was then used to calculate the overall plant length.

#### 2.3.6 Sugar beet green parts length

To determine the length of the green part, the length of the cut leaf was measured. The average of 20 plants was then used to calculate the green part length of a plant.

#### 2.3.7 Weed counting within the frame

The percentage (%) covering area and the number of weeds were calculated by counting the weeds in 4 randomly placed 1 m^2^ frames within each parcel every 15 days and then fixed.

#### 2.3.8 Fresh and dry weight of weeds

Weeds were randomly placed within the plots as close as possible to the sugar beet harvest and then placed within four 1-m^2^ frames, which were then fixed. These were mowed, weighed for fresh weight, and their average length was measured. The weeds were then spread out in a sunny area to dry, and the dry weight of the dried weeds was weighed. Weeds present in the frames during harvest were also counted.

### 2.4 Statistical analysis

All field data were analyzed according to a randomized complete block design with four replications and evaluated separately for each year (2020 and 2021). An analysis of variance (ANOVA) was performed using SPSS (v25.0), and treatment means were compared with Tukey’s HSD test at P ≤ 0.05. For interpretability, percentage increases (relative to the weedy control) and percentage decreases (relative to the weed-free control) were summarized alongside raw means in the results tables, consistent with the presentation of yield and weed suppression metrics in the study.

## 3 Result and discussion

### 3.1 Yield and growth responses to weed control treatments

Weed control treatments significantly affected all yield components of sugar beet during both experimental years (2020–2021) (**Table 5**). The weed-free control (T2) produced the highest total yield 262.58 t ha^-1^ in 2020 and 222.34 t ha^-1^ in 2021 followed closely by the integrated treatments T7 (metamitron + one hoeing) and T8 (metamitron + two hoeings). In contrast, the weedy control (T1) exhibited the lowest yields (54.56 t ha^-1^ in 2020 and 44.44 t ha^-1^ in 2021), confirming the destructive effect of weed competition.

**Table 5:** Effects of applications on sugar beet parameters in 2020 and 2021.

Applications	The year 2020							The year 2021						
	Whole weight (Ton)	Root weight (Ton)	Leaf weight (Ton)	Root diameter (cm)	Whole length (cm)	Root length (cm)	Leaf length (cm)	Whole weight (Ton)	Root weight (Ton)	Leaf weight (Ton)	Root diameter (cm)	Whole length (cm)	Root length (cm)	Leaf length (cm)
T1	54.56 d	43.54 e	12.04 b	27.93 e	60.25 b	22.20 c	38.26 ab	44.44 d	32.96 e	11.36 c	25.79 d	57.66 b	23.49 d	34.18 a
T2	262.58 a	215.44 a	40.05 a	50.21 a	69.34 ab	31.30 a	38.16 ab	222.30 a	191.03 a	29.14 a	43.58 a	67.90 a	34.50 a	33.40 a
T3	94.84 d	76.11 de	18.84 b	34.19 de	71.03 a	26.76 abc	44.24 a	91.69 cd	76.05 de	15.75 bc	34.20 b	65.15 ab	28.93 bc	36.10 a
T4	89.44 d	72.96 de	17.04 b	33.10 de	67.75 ab	24.70 bc	43.09 a	78.64 cd	64.46 de	13.16 c	30.10 cd	64.66 ab	25.54 cd	39.15 a
T5	107.78 cd	97.31 cde	16.03 b	35.55 d	69.14 ab	26.63 abc	42.51 a	96.08 cd	78.86 de	18.45 abc	34.15 b	67.44 a	28.23 bcd	39.01 a
T6	122.29 bc	105.86 cd	16.31 b	37.49 cd	65.33 ab	28.19 ab	37.11 ab	122.63 bc	103.28 bcd	19.35 abc	37.30 ab	67.68 a	31.90 ab	35.53 a
T7	191.53 ab	167.79 ab	23.23 a	44.34 ab	64.84 ab	29.55 ab	35.29 ab	167.85 ab	142.88 abc	25.65 ab	41.39 ab	67.14 a	31.90 ab	35.24 a
T8	173.70 bc	153.79 bc	19.91 b	43.60 bc	64.18 ab	29.68 ab	34.63 ab	173.25 ab	153.23 ab	20.15 abc	43.21 a	64.69 ab	30.83 ab	33.86 a
T9	125.10 bcd	109.63 bcd	15.58 b	39.23 bcd	62.54 ab	27.85 ab	34.69 ab	119.59 bc	101.93 cd	16.99 bc	37.23 ab	63.56 ab	29.83 abc	33.75 a
T10	114.41 bcd	99.39 cde	15.02 b	37.19 d	61.10 ab	26.74 abc	34.49 ab	106.88 c	90.79 d	15.86 bc	34.61 b	62.80 ab	28.45 bcd	34.23 a
T11	107.89 cd	94.44 cde	13.44 b	36.96 d	61.09 ab	26.91 abc	34.18 ab	97.20 cd	83.36 de	13.95 c	33.88 b	60.79 ab	28.49 bcd	32.20 a
T12	101.70 cd	88.37 de	14.34 b	35.66 d	59.14 b	29.13 ab	30.01 b	97.99 cd	84.32 d	14.01 c	34.64 b	61.34 ab	28.24 bcd	33.35 a
TUKEY	78.71	60.04	16.95	6.38	10.63	5.23	10.59	57.55	50.96	10.70	7.57	8.35	5.22	7.85

*There is no statistical difference between applications containing the same letters in the same columns. (TUKEY> 0.05).

As shown in **Table 6**, the percentage increase in total yield relative to T1 was remarkable 251.0% (T7) and 218.3% (T8) in 2020, and 277.7% (T7) and 289.9% (T8) in 2021. Similarly, root yield increased by 285.4% (T7) and 364.9% (T8) over the weedy control, while reductions compared with the weed-free control (T2) were limited to only 22.1–28.6%. These results demonstrate that maintaining weed-free conditions during the first 6–8 weeks after emergence the critical period of weed competition (CPWC) is essential for preserving yield potential.

**Table 6:** Sugar beet total weight and root weight yield values and percentage increase and decrease rates (2020 and 2021).

No	Applications	The year 2020						The year 2021					
		Whole weight (Ton)	% increase*	% decrease**	Root weight (Ton)	% increase*	% decrease**	Whole weight (Ton)	% increase*	% decrease**	Root weight (Ton)	% increase*	% decrease**
1	T1	54.56		79.22	43.54		79.79	44.44		80.01	32.96		82.74
2	T2	262.58	381.24		215.44	394.83		222.30	400.25		191.03	479.52	
3	T3	94.84	73.81	63.88	76.11	74.81	64.67	91.69	106.33	58.76	76.05	130.72	60.19
4	T4	89.44	63.92	65.94	72.96	67.57	66.14	78.64	76.96	64.63	64.46	95.56	66.25
5	T5	107.78	97.53	58.95	97.31	123.51	54.83	96.08	116.20	56.78	78.86	139.25	58.72
6	T6	122.29	124.12	53.43	105.86	143.15	50.86	122.63	175.95	44.84	103.28	213.31	45.94
7	T7	191.53	251.03	27.06	167.79	285.40	22.11	167.85	277.72	24.49	142.88	333.45	25.21
8	T8	173.70	218.35	33.85	153.79	253.23	28.62	173.25	289.87	22.06	153.23	364.85	19.79
9	T9	125.10	129.28	52.36	109.63	151.81	49.11	119.59	169.11	46.20	101.93	209.22	46.64
10	T10	114.41	109.69	56.43	99.39	128.29	53.86	106.88	140.51	51.92	90.79	175.43	52.47
11	T11	107.89	97.73	58.91	94.44	116.93	56.16	97.20	118.73	56.28	83.36	152.90	56.36
12	T12	101.70	86.39	61.27	88.37	102.97	58.98	97.99	120.51	55.92	84.32	155.80	55.86

*increase compared to weedy control.

**reduction compared to weed-free control.

The reduction in yield under uncontrolled weed growth (T1) was primarily caused by intense competition for light. nutrients, and soil moisture, which reduced leaf expansion and photosynthetic efficiency. Such limitations restricted assimilate translocation to storage roots, leading to smaller root diameters and lower root weights. These findings align with earlier studies by Güncan (2000) and Kaya (2012), who reported yield losses of 50–70% under similar conditions, and with Önen et al. (1998) and Işık and Akça (2018), who identified the CPWC of sugar beet as approximately 45–60 days after emergence.

In both years, the integrated treatments (T7, T8) achieved yields statistically similar to the weed-free contro, showing that metamitron combined with mechanical hoeing provides effective and durable weed suppression. The positive effect of hoeing can be attributed not only to weed removal but also to improvements in soil aeration, root elongation, and moisture retention. As Buzluk and Acar (2002) indicated, mechanical hoeing breaks surface crusts, improving soil oxygenation and microbial activity—factors that support greater root biomass and sucrose accumulation. Accordingly, the combination of chemical and mechanical control reduced herbicide dependence and labor requirements while maintaining yield stability, exemplifying a practical model of integrated and sustainable weed management (Işık and Akça, 2018).

### 3.2 Weed density, cover, and biomass suppression (based on **Tables 7**, **8**)

Weed density, cover percentage, and biomass values varied markedly among treatments in both years (**Tables 7**, **8**). In 2020, the weedy control (T1) showed the highest weed density (70.69 plants m^-2^) and cover (19.75%). The integrated treatments T7 (metamitron + one hoeing) and T8 (metamitron + two hoeings) significantly reduced these parameters to 16.31 and 8.31 plants m^-2^, with corresponding weed covers of 13.00% and 9.75%, respectively. These reductions represent 76.9% and 88.2% decreases in density relative to T1.

**Table 7:** Effect of applications on weed count parameters in the experimental fields (2020-2021).

Applications	The year 2020				The year 2021			
	Coverage area (%)	% Effect	Number of Plants (number/m^2^)	% Effect	Coverage area (%)	% Effect	Number of Plants (number/m^2^)	% Effect
T1	19.75 d		70.69 e		17.25 d		39.31 d	
T2	0.00 a	100.00	0.00 a	100.00	0.00 a	100.00	0.00 a	100.00
T3	22.00 e	-11.39	61.88 de	12.47	14.38 bcd	16.67	35.69 cd	9.22
T4	14.38 bcd	27.22	35.06 b	50.40	12.38 bcd	28.26	26.00 abcd	33.86
T5	13.00 bc	34.18	56.88 cde	19.54	12.25 bcd	28.99	20.38 abcd	48.17
T6	16.63 cde	15.82	13.19 ab	81.34	16.38 d	5.07	35.38 cd	10.02
T7	13.00 bc	34.18	16.31 ab	76.92	9.50 bc	44.93	7.06 ab	82.03
T8	9.75 b	50.63	8.31 ab	88.24	9.00 b	47.83	10.81 abc	72.50
T9	17.13 cde	13.29	29.31 abcd	58.53	15.63 cd	9.42	29.81 bcd	24.17
T10	17.13 cde	13.29	23.94 abc	66.14	13.63 bcd	21.01	17.75 abcd	54.85
T11	19.25 de	2.53	31.13 abcd	55.97	17.25 d	0.00	22.06 abcd	43.88
T12	18.50 cde	6.33	40.69 bcde	42.44	11.75 bcd	31.88	15.88 abcd	59.62
TUKEY	5.83		33.10		6.50		26.38	

*There is no statistical difference between applications containing the same letters in the same columns. (TUKEY> 0.05).

**Table 8:** Effect of applications on weed harvest parameters in the experimental field (2020 and 2021).

Applications	The year 2020								The year 2021							
	Fresh weight (g)	% effect	Dry weight (g)	% effect	Weed height (cm)	% effect	Number of weeds (number/m^2^)	% effect	Fresh weight (g)	% effect	Dry weight (g)	% effect	Weed height (cm)	% effect	Number of weeds (number/m^2^)	% effect
T1	968.56 e		607.38 e		89.09 d		70.81 e		1944.06 c		1490.19 c		104.08 bc		43.56 cd	
T2	0.00 a	100.00	0.00 a	100.00	0.00 a	100.00	0.00 a	100.00	0.00 a	100.00	0.00 a	100.00	0.00 a	100.00	0.00 a	100.00
T3	860.00 de	11.21	506.31 de	16.64	87.48 d	1.82	62.19 cd	12.18	1367.13 bc	29.68	1046.31 bc	29.79	103.83 bc	0.24	32.38 bcd	25.68
T4	662.97 cde	31.55	428.97 cde	29.37	83.06 d	6.77	35.31 bcd	50.13	884.63 ab	54.50	739.50 abc	50.38	95.67 bc	8.08	33.19 bcd	23.82
T5	545.81 bcde	43.65	300.50 bcd	50.52	84.86 d	4.76	67.31 de	4.94	940.25 abc	51.63	704.69 abc	52.71	98.57 bc	5.29	28.81 bcd	33.86
T6	210.94 abc	78.22	150.97 abc	75.14	69.35 cd	22.16	14.19 ab	79.96	997.19 abc	48.71	781.81 abc	47.54	97.44 bc	6.37	32.38 bcd	25.68
T7	86.65 ab	91.05	59.90 ab	90.14	44.48 bc	50.07	17.15 ab	75.79	1139.85 bc	41.37	880.75 bc	40.90	100.60 bc	3.34	35.96 bcd	17.46
T8	37.69 a	96.11	22.06 ab	96.37	34.18 bc	61.63	10.25 ab	85.53	860.25 ab	55.75	663.56 ab	55.47	93.76 bc	9.91	29.81 bcd	31.56
T9	396.50 abcd	59.06	259.25 abcd	57.32	68.55 cd	23.06	29.69 abc	58.08	739.00 ab	61.99	482.50 ab	67.62	78.44 bc	24.63	46.88 d	-7.60
T10	382.88 abcd	60.47	233.81 abcd	61.50	66.06 cd	25.85	24.75 ab	65.05	665.25 ab	65.78	437.69 ab	70.63	77.68 b	25.36	35.88 bcd	17.65
T11	328.31 abc	66.10	176.00 abc	71.02	71.33 cd	19.94	31.38 abc	55.69	650.44 ab	66.54	456.69 ab	69.35	94.44 bc	9.26	18.75 ab	56.96
T12	335.13 abc	65.40	178.19 abc	70.66	62.44 cd	29.91	40.94 bcde	42.19	865.88 ab	55.46	694.13 abc	53.42	113.45 c	-9.00	21.38 abc	50.93
TUKEY	500.24		288.99		27.76		33.08		1054.49		806.00		35.35		23.68	

*There is no statistical difference between applications containing the same letters in the same columns. (TUKEY> 0.05).

In 2021, weed suppression patterns were consistent with those observed in 2020. Weed density decreased markedly from 39.31 plants m^-2^ in the weedy control (T1) to 7.06 plants m^-2^ in T7 and 10.81 plants m^-2^ in T8, corresponding to reductions of 82.0% and 72.5%, respectively. Similarly, weed cover declined from 17.25% in T1 to 9.5% in T7 and 9.0% in T8. Herbicide-only treatments (T3–T6) resulted in moderate weed suppression, whereas mechanical methods, including hand hoeing and power tillage (T9–T12), achieved higher control efficiencies ranging from 60% to 75%.

Weed biomass data in **Table 8** further confirm the superior performance of integrated approaches. In 2020, the fresh weed weight decreased from 968.56 g m^-2^ (T1) to 37.69 g m^-2^ (T8) a 96.1% reduction, and the dry weed weight dropped from 607.38 g m^-2^ to 22.06 g m^-2^, representing a 96.37% reduction. Similarly, in 2021, the fresh and dry weights were reduced by 55.75–55.47% under T8 compared to the weedy control. These results indicate that combined chemical and mechanical control achieved nearly complete suppression of both annual and perennial weed species throughout the growing period.

The close association between weed biomass and yield response observed here supports earlier findings that sugar beet yield is inversely proportional to weed density and biomass (Güncan, 2000; Kaya, 2012; Işık and Akça, 2018). The yield increments shown in **Table 6** align directly with the reductions in dry weed weight recorded in **Table 8**, confirming that maintaining low weed biomass (<100 g m^-2^) during the first 6–8 weeks after emergence is crucial to prevent irreversible yield losses.

These results also demonstrate the time-dependent efficacy of integrated control: metamitron provided early suppression, while subsequent hoeing prevented late-season weed emergence.

Such combined approaches reduced the need for repeated herbicide applications and minimized environmental load, consistent with the principles of Integrated Weed Management (IWM). Comparable results were reported by Berg et al. (2025) who observed >90% weed-biomass reductions with herbicide + mechanical systems in sugar beet. Overall, **Tables 7**, **8** confirm that metamitron + hoeing provided the most stable and sustainable control, achieving over 80–90% weed suppression while maintaining yield levels statistically similar to the weed-free control (T2). This reflects the higher weed control efficacy of mechanical-chemical weed control combinations in this study compared to herbicide application alone. Other studies confirm the high weed control efficacy of mechanical-chemical combinations in sugar beet (Machleb et al., 2021; Gerhards et al., 2024; Parasca et al., 2024).

Weed presence can account for approximately 31% of yield losses in cultivated plants (Oerke et al., 1994), and weeds remain one of the most significant biotic constraints on crop productivity worldwide (Savary et al., 2019). It has been reported that weeds are the direct cause of a 5.8% decrease in yield per unit area in sugar beet (Güncan, 2000). In the study, although the T7 (700 g L^-1^ metamitron + one hand hoe) application gave the best results in 2020 compared to the T2 (weed-free control) application, according to the increase and decrease rate in root yield, the yield loss compared to T2 (weed-free plot) was 22.11%. In T1 (weedy control), the loss was 79.79%. In 2021, even though T8 (700 g L^-1^ metamitron + two hand hoeing) gave the best results compared to T2 (weed-free control), there was a 19.79% yield loss, while in T1 (weedy control), the loss was 82.74%. As a result of the study, it was determined that weed directly reduced sugar beet yield by 19.79% at the lowest and 82.74% at the highest. The greatest significant root length, root diameters and root yield plant were recorded when hand weeding twice followed by Betanal MaxxPro without significant differences (Abd El Lateef et al., 2021).

The most successful application in 2020, based on root weight, was T2 (weed-free control), with 215.44 tons. The second-highest yields were obtained from T7 (700 g L^-1^ metamitron + one hand hoe) with 167.79 tons, and T8 (700 g L^-1^ metamitron + two hand hoe) with 153.79 tons. While the third best yielding applications were T5-T6-T10-T11-T12, the lowest root yield was 43.54 tons in the T1 (weed control) application.

The application with the highest root weight in 2021 was T2 (weed-free control) with 191.03 tons. The second-best application was T8 (700 g L^-1^ metamitron + hand hoeing twice) with 153.22 tons and T7 (700 g L^-1^ metamitron + hand hoeing once) with 142.88 tons. The third application was T6-T9, the fourth was T3-T5-T10-T11-T12, the fifth was T4, and the application with the lowest weight was T1 (weedy control) with 32.96 tons.

The effects of weed competition on sugar beet yield were investigated in Tokat Province. The study determined that weed competition begins immediately after emergence. Even after 10 days, a 12.4% difference was reported between plots that were completely weeded and those that were left with weeds. Depending on ecological conditions, economic damage occurs 10–20 days after the 2–3 leaf stage. As the duration of weed competition increases, sugar beet root yield may decline by 10.53% to 88.33% compared to weed-free conditions. Therefore, early weed control, particularly before or shortly after sowing, has been strongly recommended to prevent severe yield losses (Önen et al., 1998; Işık and Akça, 2018). In the study, the best application after weed-free control in both years was obtained when pre-emergence herbicide and hand hoeing were used once or twice. Although pre-emergence application alone is not sufficient to increase yield value, the necessity of starting the control before emergence and yield losses is parallel to the study conducted in Tokat province.

In the study investigating the effects of weed control methods on yield and quality, the highest sugar beet yield of 59480 kg ha-1 was obtained from the application of full-area weed spraying with low dose post-emergence herbicide + power tiller (herbicide + hoe). It was reported that the lowest sugar beet yield was obtained from the rotary cultivator machine with 52600 kg ha-1 (Buzluk and Acar, 2002). In the study conducted, although the use of pre-emergence herbicide and two power tiller cycles increased yields in both years compared to T1 (weedy control), it was not the most successful result. The different herbicides used, their use at different times, and the different ecological values may have contributed to the different results.

According to the results of the research on the possibilities of reducing herbicide use in sugar beet cultivation, 2 times hand hoeing + 2 times tractor hoeing provided 89.9%, 3 times low-dose post-emergence full-area herbicide application 88.4%, 1 time low-dose post-emergence band herbicide + thinning + 1 time tractor hoeing 85.7%, 1 time low-dose post-emergence band herbicide + 2 times tractor hoeing 78.4%, 3 times low-dose post-emergence band herbicide + 1 time tractor hoeing 76%, 2 times low-dose post-emergence band herbicide + 1 time tractor hoeing provided 68% weed control. In terms of root and sugar yields, the differences between them were not significant when compared with the control (58.98 and 9.77 t ha^-1^), but the best result was 2 times tractor hoeing + thinning (58.07 and 9.63 t ha^-1^). Root and sugar yields of other experimental subjects were statistically lower than those of the control. The results showed that in arid and semiarid regions, two applications of tractor hoeing combined with thinning, as well as fully mechanized systems involving low-dose post-emergence band herbicide applications integrated with tractor hoeing, provided superior weed control and yield performance compared to other band herbicide combinations. Moreover, such mechanized integrated systems resulted in up to a 70% reduction in herbicide use per unit area when compared with repeated full-field post-emergence applications (Kaya, 2012; Machleb et al., 2021). In our study, it was observed that when pre-emergence herbicides were used together with mechanical control, they controlled weeds and resulted in significant yield increases.

Field experiments were conducted during the seasonal growth periods of sugar beet in Kayseri, Türkiye, in 2012 and 2013 to evaluate the critical weed control period (CPWC). Data were collected during periods of increased weed disturbance and, for comparison, during weed-free periods. In both years, relative root yield of sugar beet decreased during periods of greater weed disturbance and increased during periods of greater weed-free periods. In 2012, CPWC ranged from 122 to 595 GDD (growing degree days), corresponding to 12 to 46 days after crop emergence (DAE). The following year, CPWC was found to range from 82 to 735 GDD (8 to 54 DAE) based on a 5% acceptable yield loss. To prevent a loss of more than 5% in sugar beet root yield, weed-free conditions must be established from the first week after crop emergence and maintained for up to nine weeks (Işık and Akça, 2018). These results could help sugar beet producers significantly reduce their costs and increase the effectiveness of their weed management programs. These results are similar to our study, which found that early weed control and a period of weed-free cultivation increased yields.

## 4 Conclusion

This two-year field study conducted in Yeni Village, Doğanşehir District, Malatya Province (Türkiye) clearly demonstrated that weed management is a decisive factor influencing sugar beet yield and growth performance. The findings revealed that pre-emergence herbicide (700 g L^-1^ metamitron) alone was insufficient to provide season-long control. However, when combined with one or two hand hoeings (T7 and T8), it ensured effective and consistent weed suppression comparable to the weed-free control. These integrated treatments substantially increased both root and total yields while reducing weed density and biomass by more than 90%. Plots left untreated (T1) experienced severe yield losses of up to 365%, highlighting the destructive impact of uncontrolled weed competition during early growth stages. The integrated approaches (T7 and T8) not only enhanced crop performance but also delivered environmental and agronomic advantages by reducing herbicide dependence, limiting weed resurgence, and maintaining yield stability under field conditions. Overall, the results confirm that Integrated Weed Management (IWM), combining chemical and mechanical methods, represents the most effective and sustainable approach for sugar beet production in Türkiye and similar agroecological regions. Future research should focus on optimizing hoeing frequency, assessing the economic feasibility of integrated systems, and exploring their compatibility with reduced herbicide doses to promote environmentally sound and economically resilient weed management practices that support long-term agricultural sustainability.

## Data Availability

The original contributions presented in the study are included in the article/supplementary material. Further inquiries can be directed to the corresponding author.
